# Understanding the Regioselectivity and the Molecular Mechanism of [3 + 2] Cycloaddition Reactions between Nitrous Oxide and Conjugated Nitroalkenes: A DFT Computational Study

**DOI:** 10.3390/molecules27238441

**Published:** 2022-12-02

**Authors:** Ewa Dresler, Aneta Wróblewska, Radomir Jasiński

**Affiliations:** 1 Łukasiewicz Research Network, Institute of Heavy Organic Synthesis “Blachownia”, Energetyków 9, 47-225 Kędzierzyn-Koźle, Poland; 2 Centre of Molecular and Macromolecular Studies, Polish Academy of Sciences, Sienkiewicza 112, 90-363 Lodz, Poland; 3 Institute of Organic Chemistry and Technology, Cracow University of Technology, Warszawska 24, 31-155 Cracow, Poland

**Keywords:** [3 + 2] cycloaddition, nitrous oxide, nitroalkenes, molecular mechanism, molecular electron density theory, conceptual density functional theory

## Abstract

Regiochemical aspects and the molecular mechanism of the [3 + 2] cycloaddition between nitrous oxide and conjugated nitroalkenes were evaluated on the basis of the wb97xd/6-311 + G(d) (PCM) computational study. It was found that, independently of the nature of the nitroalkene, all considered processes are realized via polar, single-step mechanisms. All attempts at the localization of hypothetical zwitterionic intermediates were unsuccessful. Additionally, the DFT computational study suggested that, in the course of the reaction, the formation of respective Δ^2^-4-nitro-4-R_1_-5-R_2_-1-oxa-2,3-diazolines was preferred from the kinetic point of view.

## 1. Introduction

Five-membered heterocycles including nitrogen atom(s) are an important part of many molecular systems with a valuable role in the fields of pharmacy and biotechnology [1,2,3]. The introduction of nitro groups for these types of molecules increases the potential activity of the considered molecules [4]. Additionally, the nitro group opens a wide range of possibilities for the further functionalization of amines [5], nitrile N-oxides [6], oximes [7], nitronic esters [8], and many others [9]. Thus, the question of the preparation of nitro-functionalized five-membered heterocycles has attracted the attention of many researchers around the world.

**Scheme 1:**
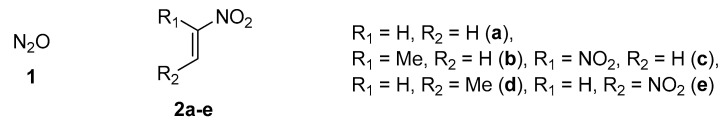
Components for the model of [3 + 2] cycloaddition reactions.

This work consisted of the continuation of our systematic studies regarding the [3 + 2] cycloaddition (32CA) processes involving conjugated nitroalkenes. Previously, we experimentally and theoretically analyzed 32CAs with the participation of nitrones [10,11], azomethine ylides [12], thiocarbonyl ylides [13], diazo compounds [14], nitrile N-oxides [15,16], and azides [17]. In the framework of this paper, we decided to shed light on the potential regioselectivity and molecular mechanism of 32CA with the participation of nitrous oxide (**1**) as a three-atom component (TAC) [18]. As 2π components within this study, we tested nitroethene (**2a**) and its analogs with different types and positions of the functionalization (Scheme 1). In particular, we selected different types of methyl- and nitro-substituted analogs of nitroethene (**2b–e**).

It must be underlined that the mechanistic aspects of this type of reaction cannot be predicted a priori, because in recent years many examples of stepwise 32CAs with biradical or zwitterionic intermediates have been detected [19]. For this, study we applied results derived from DFT calculations. We hope that our research will be helpful for further experimental studies in the mentioned area.

## 2. Results and Discussion

**Scheme 2:**
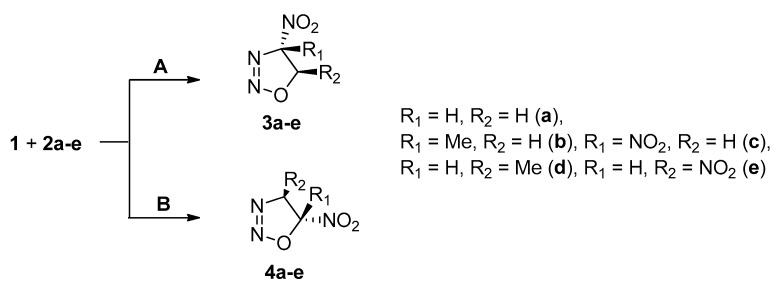
Theoretically possible regioisomeric channels of the [3 + 2] cycloaddition with the participation of nitrous oxide (**1**) and conjugated nitroalkenes (**2a–e**).

The title reactions can be theoretically realized according to two competitive, regioisomeric cycloaddition channels: A, leading to Δ^2^-4-nitro-4-R_1_-5-R_2_-1-oxa-2,3-diazolines (**3a–d**); and B, leading to Δ^2^-5-nitro-4-R_1_-5-R_2_-1-oxa-2,3-diazolines (**4a–d**) (Scheme 2). In the case of reaction with the participation of 1,2-dinitroethene, only one reaction channel (**3e = 4e**) is possible due to the symmetrical nature of the 2π component.

We started our study from the analysis of the nature of intermolecular interactions between cycloaddition components. For this purpose, conceptual density functional theory (CDFT) methodologies were applied based on the results derived (according to Domingo’s recommendations [20,21,22]) from B3LYP/6-31G(d) calculations. In the framework of CDFT, it is generally known that values of electronic chemical potential μ can estimate the direction of the electron density flux between components of bimolecular reactions. In the case of all considered reactions, the predicted electron flux should be observed from nitrous oxide (**1**) to the respective nitroalkene. Therefore, in Domingo’s terminology, all considered cycloadditions should be classified as forward electron density flux (FEDF) processes [22]. Next, estimated values of global electrophilicity show without any doubts that all analyzed reactions should be treated as polar, due to their respective Δω indices (Table 1).

For the exploration of the energetic profiles of the aforementioned processes, the wb97xd functional with the 6-311 + G(d) basis set was applied. A similar level of theory was very recently used for the exploration of different types of bimolecular processes, such as [3 + 2] cycloadditions [14,23] and [4 + 2] cycloadditions [24,25].

Our study started from the model process involving nitroethene (**2a**) in the simulated toluene solution. The wb97xd/6-311 + G(d) (PCM) calculations showed that the nature of the energetic profiles was qualitatively similar in the case of both considered channels. In particular, between the valleys of the initial molecular system (**1** + **2a**) and the valleys of the respective cycloadducts, only two critical points were located. These points are connected with the existence of (firstly) pre-reaction molecular complexes (MCs) and (in the second stage) transition states (TSs).

**Figure 1:**
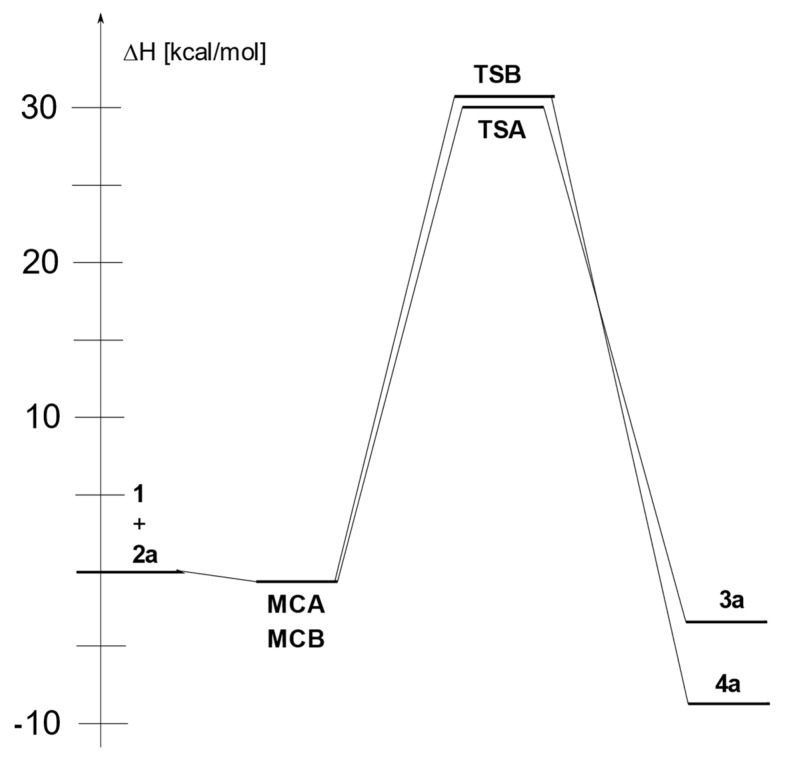
Enthalpy profile for the [3 + 2] cycloaddition between nitrous oxide (**1**) and nitroethene (**2a**) in the toluene solution according to the wb97xd/6-311 + G(d) (PCM) calculations.

**Figure 2:**
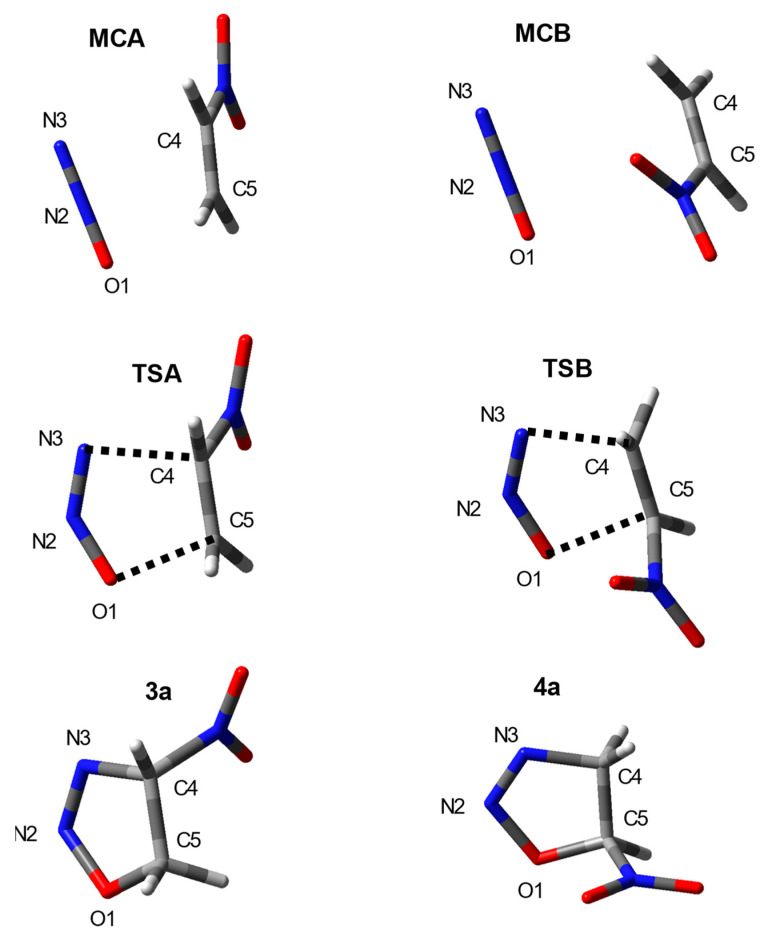
Views of key structures for [3 + 2] cycloaddition between nitrous oxide (**1**) and nitroethene (**2a**) in the toluene solution according to the wb97xd/6-311 + G(d) (PCM) calculations.

Intermolecular interactions between the initial molecules in the early reaction stages led to the formation of molecular complexes (MCs) (Figure 1). This was associated with a reduction in the enthalpy of the reaction system by about 0.2–0.4 kcal/mol (Table 2). However, the change in the entropy stimulated the positive values of the respective Gibbs free energies. Thus, MCs cannot exist in the reaction environment as stable intermediates. At this stage, no new sigma bonds are formed. All key distances exist beyond the area typical of the new sigma bonds in transition states (r > 3.3 Å). Interestingly, on both reaction paths, the addends adopt the same orientation, causing further regio-orientation within the transition state (Figure 2). Thus, optimized structures should be treated as orientation complexes. Next, the GEDT values show without any doubts that the localized structures do not exhibit the nature of charge-transfer complexes (Table 3). Similar types of pre-reaction complexes were identified very recently in different bimolecular processes [26,27,28,29].

The conversion of MCs, independently of the reaction paths, led directly to the area of the respective transition state (TSA and TSB for paths A and B, respectively). This was accompanied by a substantial increase in the enthalpy of the reaction system, by about 30 kcal/mol. However, from the kinetic point of view, cycloaddition path A is favored, so the formation of Δ^2^-4-nitro-1-oxa-2,3-diazoline (**3a**) is more probable in the course of the reaction.

**Scheme 3:**
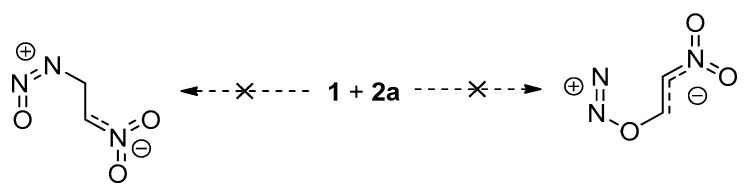
Hypothetical zwitterionic intermediates in the reaction between nitrous oxide (**1**) and nitroethene (**2a**).

Within TSs, key interatomic distances were reduced substantially. It is interesting that the kinetically favored TSA is less synchronic than TSB, as evident in the light of the Δl values. The formation of new sigma bonds was associated with the flux of the electron density. The obtained results show that the direction of this flux is compatible with the earlier CDFT prediction. On the other hand, values of global electron density transfer (GEDT) for the considered TSs were similar (GEDT = 0.18e and 0.17e for TSA and TSB, respectively). This observation confirms the polar nature of the analyzed process. The IRC calculations connect the localized TSs with valleys of the respective MCs from the one side, and with valleys of the respective cycloadducts from the other side. This excludes the possibility of a stepwise mechanism of the cycloaddition. All attempts at the optimization of hypothetical zwitterionic intermediates (Scheme 3) were unsuccessful.

Next, we used a similar approach to analyze other 32CAs with the participation of different type-1 and -2 substituted analogs of nitroethene (**2a**). It was found that, independently of the position of the substituent and its nature, cycloaddition processes involving all conjugated nitroalkenes were realized via single-step mechanisms. Additionally, all of the considered reactions should be treated as polar. The polar nature of transition states is increased to some extent with the increase in the polarity of the solvent. However, these changes are not sufficient for the enforcement of possible stepwise, zwitterionic mechanisms. In conclusion, in light of our results, the proposed mechanism can be treated as general for the 32CA processes between nitrous oxide and conjugated nitroalkenes.

## 3. Computational Details

All quantum chemical calculations were performed using the ‘Prometheus’ infrastructure, which was shared by the ACK ‘Cyfronet’ in Cracow. The wb97xd method [30] and the 6-311 + G(d) basis set were implemented in the Gaussian 09 package [31].

All critical structures were optimized using the Berny algorithm and were characterized by frequency calculations. It was found that all addends, molecular complexes (MCs), and products had positive Hessian matrices, whereas all transition states (TSs) had one negative eigenvalue in their Hessian matrices. For all transition states, intrinsic reaction coordinate (IRC) calculations were performed. The solvent effect was implemented using the polarizable continuum model (PCM) [32]. Global electron density transfer (GEDT) between substructures of the transition state [33] was calculated according to the following equation:

GEDT = ΣqA

where qA is the net Mulliken charge, and the sum is taken over all of the atoms of the nitroalkene.

All visualizations were prepared using GaussView [34].

In turn, the σ-bond development (l) indices were designated based on the following formula [10]:

$$\;l_{X-Y}=1-\frac{r{\;}_{X-Y}^{TS}\;-\;r{\;}_{X-Y}^{P}}{r{\;}_{X-Y}^{P}}$$

The results of the quantum chemical calculations are shown in Table 1 and Table 2.

CDFT reactivity indices [20,21,22] were calculated at the B3LYP/6-31G(d) computational level because the electrophilicity scale is established at that level. The global electrophilicity index (ω) is given by the expression ω = μ^2^/2η, in terms of the electronic chemical potential (μ) and chemical hardness (η). Both quantities may be approached in terms of the one-electron energies of the frontier molecular orbitals HOMO and LUMO, as μ ≈ (E_HOMO_ + E_LUMO_)/2 and η ≈ E_LUMO_ − E_HOMO_, respectively.

## 4. Conclusions

Our computational wb97xd/6-311 + G(d) (PCM) study shed light on the probable regioselectivity as well as the molecular mechanisms of [3 + 2] cycloaddition processes with the participation of nitrous oxide as a TAC, along with different types of conjugated nitroalkenes as 2π components. In particular, the obtained results in the framework of the conceptual DFT approach indicate the polar, FEDF-type nature of all of the analyzed reactions. The analysis of the global electron density transfer between substructures of localized transition states led to similar conclusions. All localized transition states exhibited an asynchronous nature. However, the influence of the polarity of the solvent on the synchronicity of TSs was not high. All attempts at the optimization of hypothetical zwitterionic intermediates were unsuccessful. In light of our results, the proposed one-step polar mechanism can be treated as general for the 32CA processes between nitrous oxide and conjugated nitroalkenes. Experimental aspects of the title reactions will be the subject of our further studies.

## Figures and Tables

**Table 1:** Global electron properties for nitrous oxide (**1**) and conjugated nitroalkenes (**2a–e**).

	μ (eV)	η (eV)	ω (eV)	Δω (eV)
**1**	−4.92	8.79	1.37	
**2a**	−5.33	5.45	2.61	1.23
**2b**	−5.16	5.48	2.43	1.05
**2c**	−5.98	5.03	3.56	2.18
**2d**	−5.08	5.48	2.35	0.98
**2e**	−6.49	4.66	4.52	3.15

**Table 2:** Kinetic and thermodynamic description of the considered paths of [3 + 2] cycloaddition with the participation of nitrous oxide (**1**) and conjugated nitroalkenes (**2a–e**) according to the wb97xd/6-311 + G(d) (PCM) calculations.

Solvent	Transition	ΔH	ΔS	ΔG
Toluene	**1 + 2a** **→ MCA**	−0.2	−14.1	4.0
	**1 + 2a** **→ TSA**	30.3	−30.3	39.3
	**1 + 2a** **→ 3a**	−2.8	−31.0	6.4
	**1 + 2a** **→ MCB**	−0.4	−16.0	4.4
	**1 + 2a** **→ TSB**	31.2	−30.4	40.2
	**1 + 2a** **→ 4a**	−8.1	−30.8	1.1
Acetone	**1 + 2a** **→ MCA**	0.0	−11.5	3.5
	**1 + 2a** **→ TSA**	30.5	−31.2	39.8
	**1 + 2a** **→ 3a**	−3.4	−32.4	6.2
	**1 + 2a** **→ MCB**	−0.7	−18.7	4.9
	**1 + 2a** **→ TSB**	31.5	−31.4	40.9
	**1 + 2a** **→ 4a**	−9.0	−32.1	0.6
Nitromethane	**1 + 2a** **→ MCA**	0.1	−11.2	3.4
	**1 + 2a** **→ TSA**	30.5	−31.2	39.8
	**1 + 2a** **→ 3a**	−3.5	−32.4	6.2
	**1 + 2a** **→ MCB**	−0.7	−18.6	4.9
	**1 + 2a** **→ TSB**	31.6	−31.4	40.9
	**1 + 2a** **→ 4a**	−9.0	−32.2	0.5
Water	**1 + 2a** **→ MCA**	0.1	−11.2	3.4
	**1 + 2a** **→ TSA**	30.5	−31.2	39.8
	**1 + 2a** **→ 3a**	−3.5	−32.4	6.1
	**1 + 2a** **→ MCB**	−0.6	−18.7	4.9
	**1 + 2a** **→ TSB**	31.6	−31.4	40.9
	**1 + 2a** **→ 4a**	−9.1	−32.2	0.5
Toluene	**1 + 2b** **→ MCA**	−0.6	−15.0	3.9
	**1 + 2b** **→ TSA**	31.1	−31.8	40.6
	**1 + 2b** **→ 3b**	−3.8	−33.1	6.1
	**1 + 2b** **→ MCB**	−0.9	−16.5	4.0
	**1 + 2b** **→ TSB**	30.5	−31.8	40.0
	**1 + 2b** **→ 4b**	−8.7	−33.4	1.3
Toluene	**1 + 2c** **→ MCA**	−0.4	−15.5	4.2
	**1 + 2c** **→ TSA**	31.0	−33.4	40.9
	**1 + 2c** **→ 3c**	−2.3	−34.2	7.9
	**1 + 2c** **→ MCB**	−0.5	1169.2	4.8
	**1 + 2c** **→ TSB**	32.8	−33.5	42.8
	**1 + 2c** **→ 4c**	−9.7	−33.8	0.4
Toluene	**1 + 2d** **→ MCA**	−0.6	−13.8	3.5
	**1 + 2d** **→ TSA**	30.8	−30.6	40.0
	**1 + 2d** **→ 3d**	−2.5	−32.2	7.1
	**1 + 2d** **→ MCB**	−0.6	−16.3	4.3
	**1 + 2d** **→ TSB**	33.4	−30.8	42.6
	**1 + 2d** **→ 4d**	−6.8	−32.1	2.8
Toluene	**1 + 2e** **→ MC**	−0.3	−20.5	5.8
	**1 + 2e** **→ TS**	31.1	−35.3	41.7
	**1 + 2e** **→ 3e**	−4.2	−35.4	6.3

**Table 3:** Key parameters for critical structures on the considered paths of [3 + 2] cycloaddition with the participation of nitrous oxide (**1**) and conjugated nitroalkenes (**2a–e**), according to the wb97xd/6-311 + G(d) (PCM) calculations.

Solvent	Reaction	Structure	Interatomic Distances r (Å)					l_C3C4_	l_C5O1_	Δl	GEDT (e)
			O1-N2	N2-N3	N3-C4	C4-C5	C5-O1				
Toluene	**1 + 2a**	**1**	1.179	1.119							
		**2a**				1.320					
		**MCA**	1.179	1.118	3.570	1.321	3.326				
		**TSA**	1.225	1.159	2.058	1.383	1.971	0.601	0.630	0.03	0.19
		**3a**	1.364	1.226	1.471	1.517	1.438				
		**MCB**	1.179	1.118	3.558	1.321	3.315				
		**TSB**	1.217	1.165	1.919	1.382	2.079	0.690	0.488	0.20	0.19
		**4a**	1.456	1.206	1.464	1.529	1.375				
Acetone	**1 + 2a**	**1**	1.179	1.118							
		**2a**				1.321					
		**MCA**	1.178	1.118	3.407	1.321	3.294				
		**TSA**	1.224	1.158	2.068	1.383	1.970	0.594	0.633	0.04	0.18
		**3a**	1.361	1.227	1.471	1.516	1.441				
		**MCB**	1.179	1.118	3.612	1.321	3.308				
		**TSB**	1.217	1.165	1.913	1.382	2.091	0.693	0.478	0.21	0.17
		**4a**	1.458	1.206	1.464	1.528	1.375				
Nitromethane	**1 + 2a**	**1**	1.179	1.118							
		**2a**				1.321					
		**MCA**	1.178	1.118	3.406	1.322	3.294				
		**TSA**	1.224	1.158	2.069	1.383	1.970	0.594	0.634	0.04	0.18
		**3a**	1.361	1.227	1.471	1.516	1.441				
		**MCB**	1.179	1.118	3.612	1.321	3.308				
		**TSB**	1.217	1.165	1.913	1.382	2.092	0.694	0.478	0.22	0.17
		**4a**	1.458	1.206	1.464	1.528	1.375				
Water	**1 + 2a**	**1**	1.179	1.118							
		**2a**				1.321					
		**MCA**	1.178	1.118	3.398	1.322	3.296				
		**TSA**	1.224	1.158	2.069	1.383	1.970	0.594	0.634	0.04	0.18
		**3a**	1.360	1.227	1.471	1.516	1.442				
		**MCB**	1.179	1.118	3.612	1.321	3.309				
		**TSB**	1.217	1.165	1.913	1.382	2.093	0.694	0.477	0.22	0.17
		**4a**	1.459	1.206	1.464	1.528	1.375				
Toluene	**1 + 2b**	**2b**				1.325					
		**MCA**	1.178	1.119	3.305	1.325	3.329				
		**TSA**	1.225	1.158	2.071	1.388	1.985	0.598	0.617	0.02	0.21
		**3b**	1.369	1.223	1.477	1.526	1.435				
		**MCB**	1.179	1.118	3.512	1.325	3.153				
		**TSB**	1.216	1.166	1.926	1.386	2.122	0.683	0.466	0.22	0.19
		**4b**	1.442	1.211	1.463	1.527	1.383				
Toluene	**1 + 2c**	**2c**				1.317					
		**MCA**	1.181	1.118	3.981	1.317	3.150				
		**TSA**	1.227	1.152	2.098	1.389	1.886	0.556	0.686	0.13	0.34
		**3c**	1.353	1.232	1.452	1.512	1.436				
		**MCB**	1.179	1.118	3.675	1.317	3.113				
		**TSB**	1.212	1.166	1.866	1.386	2.057	0.714	0.463	0.25	0.34
		**4c**	1.528	1.193	1.451	1.536	1.338				
Toluene	**1 + 2d**	**2d**				1.326					
		**MCA**	1.180	1.118	3.468	1.327	3.174				
		**TSA**	1.224	1.161	2.041	1.389	2.015	0.607	0.608	0.00	0.20
		**3d**	1.361	1.228	1.465	1.525	1.447				
		**MCB**	1.178	1.119	3.463	1.326	3.362				
		**TSB**	1.220	1.166	1.928	1.388	2.070	0.690	0.491	0.20	0.19
		**4d**	1.460	1.206	1.472	1.536	1.371				
Toluene	**1 + 2e**	**2e**				1.318					
		**MC**	1.180	1.118	3.493	1.319	3.038				
		**TS**	1.221	1.160	1.982	1.386	1.968	0.647	0.574	0.07	0.37
		**3e**	1.412	1.217	1.465	1.523	1.380				

## Sample Availability

Not available.
